# Adherence to dehydrated bovine blood consumption and improvement in hemoglobin levels in children aged 6 to 35 months, San Juan de Lurigancho-Peru

**DOI:** 10.3389/fnut.2026.1880873

**Published:** 2026-07-21

**Authors:** Rosa Elena Cruz Maldonado, Mauricio-Alza Saby Marisol, Joe Fernando Gerónimo Huete, Miguel Angel Flores Flores

**Affiliations:** 1 Facultad de Ciencias de la Salud. Escuela de Nutrición y Dietética, Universidad Privada Norbert Wiener, Lima, Peru; 2 Facultad de Industrias Alimentarias. Escuela de Bromatología y Nutrición Humana, Universidad Nacional de la Amazonía Peruana, Iquitos, Peru

**Keywords:** anemia, bovine, children, dehydrated blood, intake

## Abstract

**Purpose:**

This study aimed to evaluate adherence to the consumption of dehydrated bovine blood through the increase in hemoglobin in children aged 6 to 35 months.

**Patients and methods:**

An intervention study was conducted on 2,221 children from 33 health facilities in the district of San Juan de Lurigancho, Peru, where the initial hemoglobin level was measured and according to the diagnosis they were divided into two groups (with anemia and without anemia), of which an educational session on topics related to anemia prevention was developed monthly, a jar of dehydrated bovine blood was delivered, and hemoglobin monitoring was conducted for 3 months.

**Results:**

A total of 2,221 children aged 6 to 35 months were included in the analysis, after excluding two cases of severe anemia. The sample was pre-dominantly composed of children aged 12–23 months (50.4%), with a slight pre-dominance of males (54.0%). Hemoglobin levels increased progressively and significantly throughout the intervention. Mean hemoglobin rose from 10.667 ± 0.673 g/dl at baseline to 11.501 ± 0.716 g/dl at month 3, representing a mean increment of + 0.834 g/dl. The Friedman test confirmed that differences across the four time points were statistically significant (χ^2^ = 2,299.50, df = 3, *p* < 0.001), and Wilcoxon *post-hoc* comparisons revealed significant increases at each monthly interval (all *p* < 0.001).

**Conclusion:**

There was a high level of significance in iron adherence in children with moderate anemia.

## 1 Introduction

One-third of the world's population is affected by anemia, with half corresponding to iron deficiency anemia, affecting 1.24 billion individuals. The most affected are children under 5 years of age, with a prevalence of 47.4%, impacting 293 million children. Among the causes of anemia, we find infectious diseases (malaria, parasitosis), nutritional deficiencies (iron, vitamins A, B12, C, and folic acid, protein-energy malnutrition), and those caused by congenital defects in hemoglobin synthesis, or frequently a combination of these factors (1).

In Peru, the prevalence of anemia in children has remained for more than 20 years at a classification of severe public health problem according to World Health Organization (WHO) criteria (2). In 2011, the prevalence was 41.6%; after 11 years of interventions by the Peruvian state, the prevalence increased in 2022 to 42.4%, and in 2024 it rose even further to 43.7% (3).

In the document “Global Targets for 2025,” the WHO recommends that all efforts for the prevention and control of anemia should be supported primarily by a diet containing adequate amounts of bioavailable iron (4), that is, heme iron, which is found only in foods of animal origin, such as liver, blood sausage, spleen, lung, kidney, guinea pig meat, beef, among others, and has an absorption rate of 20%−30%, as opposed to non-heme iron (plant-based) which is absorbed at 0 to 10% (5). Dietary diversification and improvement are an essential part of the approaches and the key to long-term success and sustainability in controlling nutritional anemia (6).

Nutritional food education comprises learning activities that facilitate the voluntary adoption of dietary behaviors to promote health and wellbeing. Therefore, providing basic information to the child's caregiver at every care opportunity becomes a priority, since the child, starting at 6 months of age, must meet their requirement for macro and micronutrients, including all hematopoietic nutrients, solely through diet during periods when they are not being supplemented with iron. Otherwise, a vicious cycle of entering and exiting anemia can easily be established while the child is in a period of high nutritional requirements, with emphasis on the first 1,000 days of life (7, 8).

A meta-analysis conducted by Iglesias et al. (9) described that nutritional interventions reduce anemia by 25 to 44% in Latin American countries. Likewise, starting at 6 months of age, complementary foods and feeding practices are especially important in determining micronutrient sufficiency in children aged 6 to 23 months, since breast milk at this infant age makes a progressively smaller contribution to nutritional needs. If the diet is insufficient, poorly balanced, and delayed, it can cause anemia. According to surveys conducted by the Centro Nacional de Alimentación, Nutrición y Vida Saludable of Instituto Nacional de Salud (CENAN/INS), a total iron consumption of 6.09 mg of iron/day was reported in children aged 6 to 11 months (55.3% of the requirement); a consumption of 9.48 mg of iron/day (86.1%) in children aged 12 to 23 months; a consumption of 11.1 mg/day (100%) in children aged 24 to 35 months; and at the urban level, consumption in children aged 6 to 35 months was 9.92 mg/day, while at the rural level it was 8.95 mg/day. As evidenced, children aged 24 to 35 months have met their daily iron requirement; however, we still find a high prevalence of anemia even in this last age group, which would indicate the need to improve the type of iron that Peruvian children are consuming. Likewise, only 44% of children aged 6 to 11 months consumed any food with heme iron: 42.3% consumed chicken, 16.6% chicken liver, 8.8% beef, 6.3% gizzard, 3.6% pork, and only 1.7% poultry blood (10). Even worse, in 2021, 51.0% of households in Peru experienced mild food insecurity and 3.5% experienced severe food insecurity (11). The highest percentage of food insecurity occurred in four regions of the Peruvian highlands: Ayacucho with 67.2%, Apurímac with 66.9%, Cusco with 64.6%, and Puno with 61.5%, regions that also have the highest prevalences of anemia (12).

The objective of the study was to evaluate adherence to dehydrated bovine blood consumption through the increase in hemoglobin in an educational intervention on the prevention and recovery from anemia in children aged 6 to 35 months, in primary care health facilities of the Redes Integradas de Salud Lima Centro of the Ministerio de Salud, in the district of San Juan de Lurigancho, Peru.

## 2 Methods

### 2.1 Research ethics

The study was given a favorable ethical opinion for conduct by the Research Ethics Committee of Universidad Privada Norbert Wiener: File No. 0582–2023 titled: “Effect of a Nutritional Educational Intervention in Boys and Girls Aged 6 to 35 Months and Pregnant Women with Anemia in San Juan de Lurigancho, Lima, 2023” Version 02 dated 05/29/2023. The study was conducted according to the guidelines laid down in the Declaration of Helsinki (13). Prior to enrollment, trained study personnel explained the study's purpose, procedures, expected benefits, and potential risks to each mother and/or caregiver in Spanish, using accessible, non-technical language appropriate for the local population. Caregivers were informed that participation was voluntary, that they could withdraw their child from the study at any time without any effect on their access to routine health services, and that all collected data would be kept confidential and used solely for research purposes. Each participant entered the study after signing informed consent, where the health benefits and risks of participating in the study were explained. As participants were children under 35 months of age, child assent was not applicable. No financial incentive was provided to participants. Each participant's name was coded to maintain anonymity.

### 2.2 Study procedures

**Figure 1:**
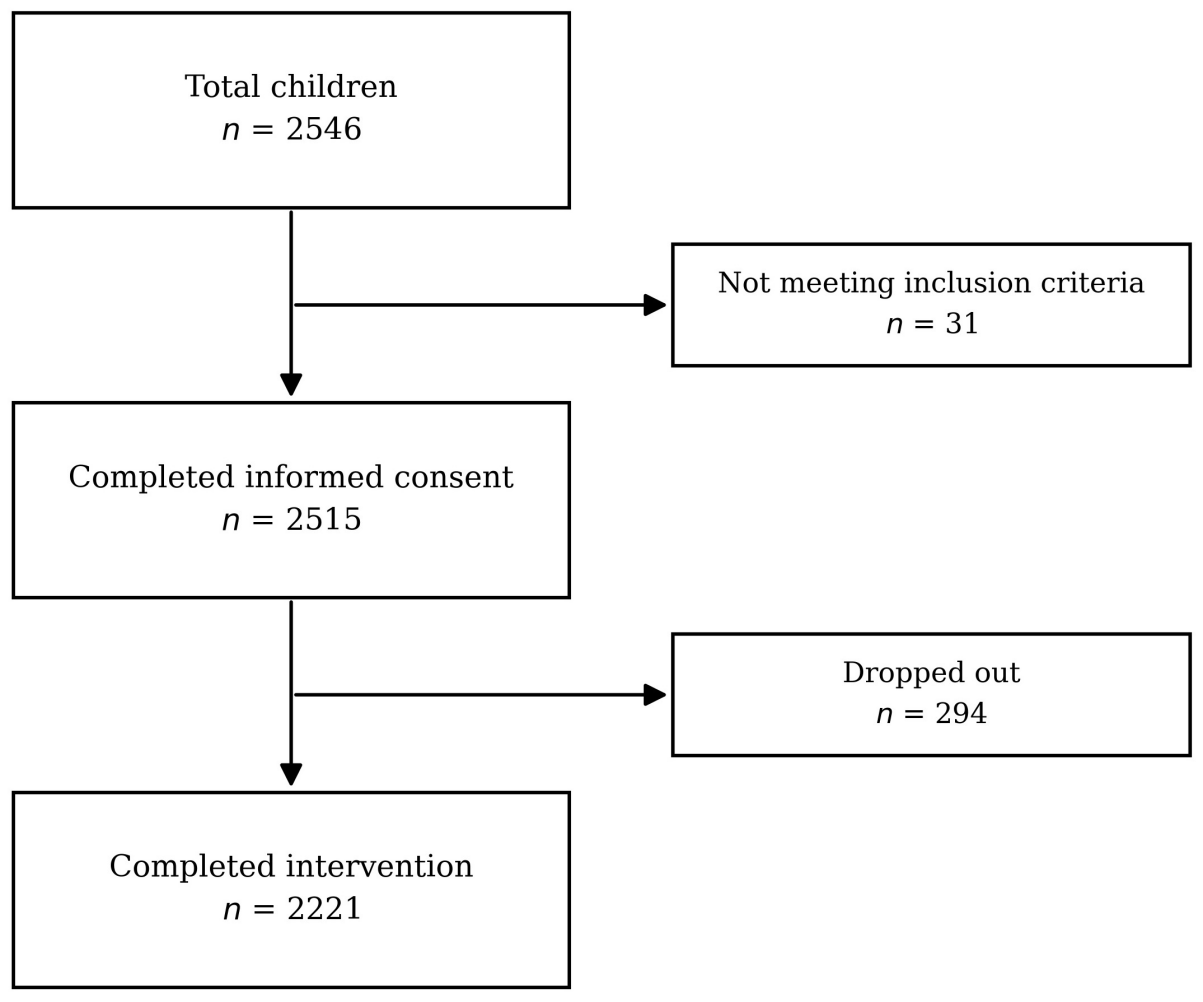
Participant flow diagram.

This was a quasi-experimental, single-group, pre-post intervention study without a comparison group with 2,546 children aged 6 to 35 months were recruited between the months of August and December 2023, from the 33 health facilities in the district of San Juan de Lurigancho, belonging to DIRIS Lima Centro (Figure 1).

DIRIS Lima Centro was requested to provide a list of children aged 6 to 35 months who had been diagnosed with anemia or with a normal hemoglobin level borderline for anemia, with a maximum age of 30 days prior to the intervention in primary care health facilities in the district of San Juan de Lurigancho. Due to the low number of children with the required hemoglobin level, a baseline hemoglobin measurement was conducted until the sample was completed (11.0 to 11.5 mg/dl). Children with borderline normal hemoglobin were intentionally included alongside children with anemia, as this group is considered at elevated risk of progressing to anemia and was therefore a relevant target for the preventive component of the intervention, in addition to its therapeutic component for children already presenting with anemia.

Mothers and/or caregivers signed informed consent to be invited to the first nutritional food education session. Children who attended the first session with their mothers and/or caregivers became part of the intervention.

Monthly educational sessions were held in groups of no more than 15 participants on the following topics: complementary feeding, iron-rich foods for children, handwashing, and deworming. At the end of each educational session, mothers and/or caregivers were given a 180 g jar of dehydrated bovine blood for daily consumption of 6 g for 30 days, totaling 3 months of distribution. The amount of dehydrated blood needed to meet the child's daily iron requirement (11 mg) was calculated according to the recommended intake in an intermediate category diet (10%−15% bioavailability) (14), which corresponded to a measure of 6 g daily of dehydrated bovine blood. The dehydrated bovine blood used in this intervention was obtained from a commercial supplier and provided to the research team in sealed 180 g jars; production and quality control of the product (dehydration method, moisture content, and microbiological testing) were the responsibility of the supplier and were not independently characterized by the research team.

**Figure 2:**
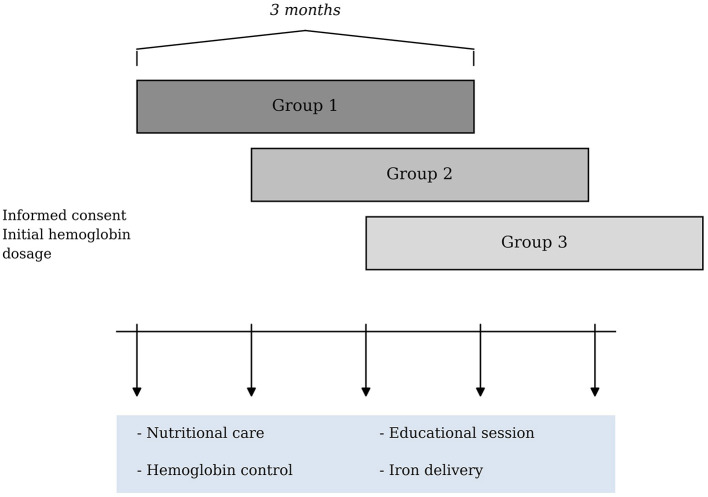
Study design.

After receiving the dehydrated blood, mothers and/or caregivers were scheduled for a nutritional consultation at the health facility before the next educational session. Adherence to dehydrated bovine blood consumption was monitored through two complementary procedures. First, each mother and/or caregiver received a 30-day take-home tracking card on which she recorded the days her child consumed the dehydrated bovine blood; when a day was inadvertently left unmarked, members of the monitoring team assisted caregivers in recalling and completing the record at the next contact. A new card was issued monthly, together with a new 180 g jar, following each educational session. Second, caregivers were gradually added to a WhatsApp group moderated by the monitoring team to monitor consumption and clarify any questions in real time; some caregivers voluntarily shared photographs of prepared meals containing dehydrated blood as informal evidence of consumption. Adverse effects associated with dehydrated bovine blood consumption were not systematically assessed using a structured tool; however, no spontaneous reports of adverse effects (e.g., gastrointestinal discomfort or taste rejection) were received through the WhatsApp monitoring channel or during monthly nutritional consultations. At the beginning of each educational session, a control hemoglobin measurement was performed in capillary blood obtained by finger-prick, using a portable hemoglobinometer (HemoCue Hb 201+, HemoCue AB, Ängelholm, Sweden; first, second, and third month of the educational intervention; Figure 2).

### 2.3 Data processing

The information captured on the registration forms was entered into a template created in Microsoft Excel 2016 and subsequently migrated to IBM SPSS version 26 (15). Age in months was categorized into three ranges: 06 to 11 months, 12 to 23 months, and 24 to 35 months. The level of anemia was classified according to internationally established parameters (16). The level of adherence was classified by taking and adapting the reference from Peruvian regulations (17). Adherence was operationally defined using hemoglobin recovery (Hb ≥ 11.0 g/dl) at month 3 as an indirect proxy of effective consumption, under the assumption that a sustained, meaningful intake of dehydrated bovine blood would be required to produce a measurable hemoglobin response. For children with anemia at baseline (mild or moderate), the primary outcome was hemoglobin recovery, defined as attaining Hb ≥ 11.0 g/dl by month 3. For children with borderline normal hemoglobin at baseline, who were not anemic, the comparable outcome instead reflects maintenance of adequate hemoglobin status rather than recovery *per se*, since this threshold was already met at enrollment; for consistency, this combined outcome is referred to as “recovery/maintenance” throughout the Results. Data collection for this intervention was limited to age, sex, health facility, and serial hemoglobin measurements; information on concurrent infections, supplementation, breastfeeding practices, household socioeconomic status, and number of children per household was not systematically collected as part of the study protocol. Children who did not complete the third hemoglobin control were excluded from the final analysis (*n* = 325 of the 2,546 originally recruited); no imputation was performed for missing follow-up data.

### 2.4 Data analysis

Descriptive statistics were reported as frequencies and percentages for categorical variables, and as means with standard deviations (SD) for continuous variables. Ninety-five percent confidence intervals (95% CI) for proportions were estimated using the normal approximation method (18).

Prior to inferential analyses, the normality of hemoglobin distributions at each time point was assessed using the Shapiro-Wilk test, which indicated significant departures from normality at all measurement points (*p* < 0.001); therefore, non-parametric tests were applied throughout. The Friedman test was selected as a non-parametric alternative to repeated-measures ANOVA, as it does not require the assumption of sphericity and is robust to departures from normality. The Friedman test operates on within-subject ranks across repeated measurements, requiring only that observations from the same child be correlated across time points (an assumption inherently satisfied by the longitudinal design) and that conditions be mutually exclusive. The Wilcoxon signed-rank test, used for *post-hoc* pairwise comparisons, similarly does not assume normality but requires that within-subject differences be symmetrically distributed around the median, a less restrictive assumption appropriate for the observed hemoglobin distributions.

To evaluate changes in hemoglobin levels over the three-month intervention period, the Friedman test was used for overall comparison across the four time points (baseline, month 1, month 2, and month 3), followed by pairwise *post-hoc* comparisons using the Wilcoxon signed-rank test. Associations between recovery status and categorical variables—including age group, sex, and baseline anemia classification—were assessed using the Pearson chi-square test (χ^2^). Adjusted residuals were calculated to identify specific cells contributing significantly to the overall chi-square statistic, with values |AR| >1.96 considered statistically significant. The strength of association between baseline anemia classification and recovery was quantified using odds ratios (OR) with 95% CI, estimated by the Woolf method, with borderline normal classification as the reference category. A transition matrix was constructed to describe changes in hemoglobin classification from baseline to month 3 (18, 19). To account for potential confounding among age, sex, and baseline anemia severity, a multivariable logistic regression model was additionally fitted with hemoglobin recovery/maintenance as the outcome, including these three covariates simultaneously. For all analysis, a significance threshold of *p* < 0.05 was applied. Statistical analyses were performed using Python (version 3.12).

## 3 Results

### 3.1 Characteristics of the study sample

A total of 2,546 children aged 06 to 35 months were recruited. During the intervention, this number decreased because they did not meet the inclusion criteria and dropped out of treatment, ultimately resulting in 2,221 with complete information (third control). In Table 1, it can be observed that during the intervention, the pre-dominant group was 12 to 23 months (50.4%), there was a slight male pre-dominance (54%), and almost 9 out of 10 children presented with mild or borderline anemia at baseline (values between 11.0 g/dl and 11.5 g/dl).

**Table 1:** Characteristics of the study sample.

Variables/categories	*n*	%	IC 95%^*^
6 to 11 months	775	34.9	(32.9–36.9)
12 to 23 months	1,119	50.4	(48.3–52.5)
24 to 35 months	327	14.7	(13.2–16.2)
Male	1,200	54.0	(52.0–56.1)
Female	1,021	46.0	(43.9–48.0)
Bordeline normal	1,012	45.6	(43.5–47.6)
Mild anemia	937	42.2	(40.1–44.2)
Moderate anemia	272	12.2	(10.9–13.6)
Total	2,221	100.0	—

^*^95% CI: 95% confidence interval calculated using normal approximation.

n, sample.

### 3.2 Evolution of hemoglobin during the intervention

The children's hemoglobin levels increased progressively and statistically significantly over the 3 months, with a total mean increase of +0.834 g/dl (Table 2). Children aged 24–35 months were four times more likely to recover than those aged 6–11 months, probably because at this age the child has greater independence, has finished complementary feeding, and is beginning to participate in family meals. No significant difference was observed between genders (Table 3).

**Table 2:** Hemoglobin evolution during the intervention.

Time point	Mean ±SD (g/dl)		Range (min–max)		Δ vs. baseline (g/dl)	*p*-value^†^	Friedman *p*-value^‡^
	Mean	SD	Min	Max			
Baseline (Hb_0_)	10.667	0.673	7.0	11.5	—	—	**< 0.001**
Month 1 (Hb_1_)	10.890	0.787	7.6	14.5	+0.222	< 0.001	
Month 2 (Hb_2_)	11.206	0.824	7.2	14.0	+0.539	< 0.001	
**Month 3 (Hb**_**3**_ **)**	11.501	0.716	7.1	14.4	+0.834	< 0.001	

^**†**^indicates from Wilcoxon signed-rank test (consecutive comparisons);

^**‡**^p-value from Friedman test for overall comparison across the four time points (χ^2^ = 2,299.50, df = 3);

All p-values < 0.001 indicate statistically significant differences.

SD, standard deviation; Δ, increment relative to baseline; Hb, hemoglobin.

**Table 3:** Recovery of hemoglobin levels by age group and gender.

Variable	*n*	Recovered *n* (%)	Not recovered *n* (%)	OR	95% CI	χ^2^	*p*-value
Age group (months)							
6–11 months (ref.)	775	667 (86.1%)	108 (13.9%)	1.00	—	**30.60**	**< 0.001**
12–23 months	1,119	1,022 (91.3%)	97 (8.7%)	**1.71**	1.28–2.28		
24–35 months	327	315 (96.3%)	12 (3.7%)	**4.25**	2.31–7.83		
Gender							
Male (ref.)	1,200	1,077 (89.8%)	123 (10.2%)	1.00	—	**0.57**	**0.451**
Female	1,021	927 (90.8%)	94 (9.2%)	**1.13**	0.85–1.49		

OR and 95% CI estimated by the Woolf method; Recovery defined as hemoglobin ≥11.0 g/dl at month 3 (SITUACION = RECOVERED); Bold p-values indicate statistically significant differences (p < 0.05).

OR, odds ratio; CI, confidence interval; χ^2^, chi-square statistic; reference category (ref.) for age group: 6–11 months; for gender: male.

Regarding hemoglobin recovery according to baseline anemia classification, statistically significant differences were observed among the three groups (χ^2^ = 72.43, df = 2, *p* < 0.001). Children classified as borderline normal at baseline achieved the highest recovery rate (95.0%), followed by those with mild anemia (88.6%) and moderate anemia (78.3%). Odds ratio analysis, using borderline normal as the reference category, revealed that children with mild anemia were 59% less likely to recover (OR = 0.41; 95% CI: 0.29–0.58), while those with moderate anemia were 81% less likely to recover (OR = 0.19; 95% CI: 0.13–0.29). These findings indicate an inverse dose-response pattern, whereby greater baseline anemia severity was associated with lower probability of hemoglobin recovery after 3 months of dehydrated bovine blood consumption (Table 4). It should be noted that the markedly higher recovery/maintenance rate observed among children with borderline normal hemoglobin at baseline partly reflects the lower threshold required for this group—simply not declining below Hb 11.0 g/dl—compared with the substantially larger hemoglobin increase required for children with mild or moderate anemia to reach the same threshold.

**Table 4:** Hemoglobin recovery according to baseline anemia classification.

Baseline classification	*n*	Recovered		Not recovered		OR (95% CI)^†^	*p*-value^‡^
		***n*** **(%)**	*AR* ^*^	***n*** **(%)**	*AR* ^*^		
Borderline normal (ref.)	1,012	961 (95.0%)	+6.87	51 (5.0%)	−6.87	1.00 —	**< 0.001**
Mild anemia	937	830 (88.6%)	−2.24	107 (11.4%)	+2.24	0.41 (0.29–0.58)	
Moderate anemia	272	213 (78.3%)	−7.07	59 (21.7%)	+7.07	0.19 (0.13–0.29)	
**Total**	2,221	2,004 (90.2%)	—	217 (9.8%)	—	—	—

^**†**^OR, odds ratio estimated by Woolf method.

Reference category: borderline normal (Hb 11.0–11.5 g/dl).

95% CI, confidence interval;

^**‡**^Overall chi-square test: χ^2^ = 72.43, df = 2, p < 0.001;

Recovery defined as hemoglobin ≥ 11.0 g/dl at month 3.

AR, adjusted residual. Values |AR| >1.96 indicate cells contributing significantly to χ^2^ (p < 0.05).

Blue shading: higher-than-expected frequency; orange shading: lower-than-expected.

*AR, adjusted residual. Values |AR| >1.96 indicate cells contributing significantly to χ^2^ (*p* < 0.05). Blue shading: higher-than-expected frequency; orange shading: lower-than-expected.

To account for potential confounding between age, sex, and baseline anemia severity, a multivariable logistic regression model was fitted with hemoglobin recovery/maintenance as the outcome. After mutual adjustment, both older age and milder baseline anemia classification remained independent predictors of recovery, with estimates closely consistent with the unadjusted analyses (age 24–35 vs. 6–11 months: adjusted OR = 3.90, 95% CI 2.10–7.22; moderate vs. borderline anemia: adjusted OR = 0.20, 95% CI 0.13–0.30), while sex remained non-significant (adjusted OR = 1.06, 95% CI 0.79–1.42, *p* = 0.688; Table 5).

**Table 5:** Transition matrix of hemoglobin classification from baseline to month 3.

Variable	aOR	95% CI	*p*-value
Age group (months)	
6–11 (ref.)	1.00	—	—
12–23	**1.71**	1.27–2.30	**< 0.001**
24–35	**3.90**	2.10–7.22	**< 0.001**
Sex	
Male (ref.)	1.00	—	—
Female	1.06	0.79–1.42	0.688
Baseline anemia classification	
Borderline normal (ref.)	1.00	—	—
Mild anemia	**0.42**	0.30–0.60	**< 0.001**
Moderate anemia	**0.20**	0.13–0.30	**< 0.001**

aOR, adjusted odds ratio, mutually adjusted for age group, sex, and baseline anemia classification in a single multivariable logistic regression model; CI, confidence interval.

Reference categories (ref.): age group, 6–11 months; sex, male; baseline anemia classification, borderline normal (Hb 11.0–11.5 g/dl).

Outcome: hemoglobin recovery (Hb ≥ 11.0 g/dl) at month 3.

Bold values indicate statistically significant associations (p < 0.05).

**Figure 3:**
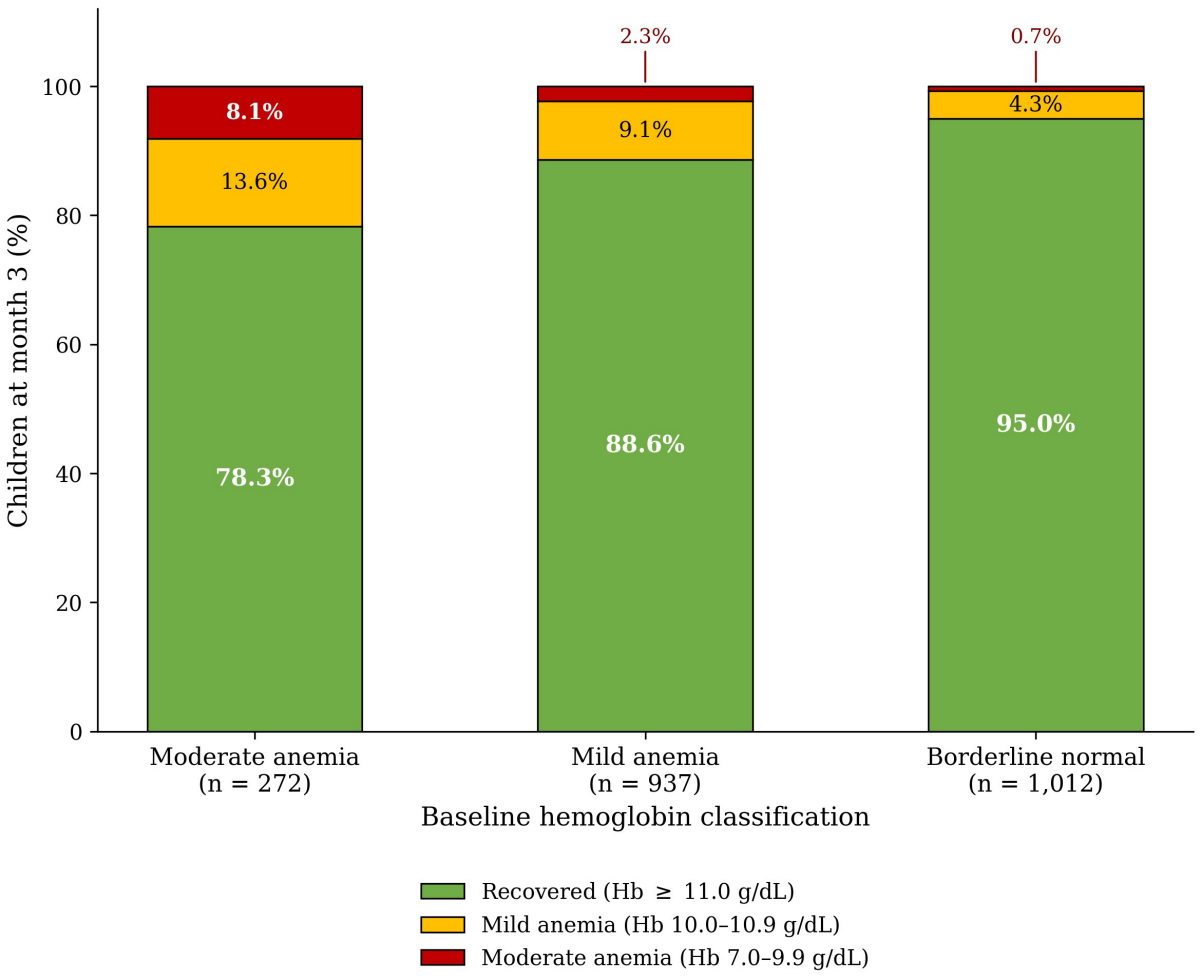
Hemoglobin classification at month 3 by baseline category.

The transition matrix revealed the changes in hemoglobin classification from baseline to month 3 across all groups. Among children initially classified with moderate anemia, 78.3% achieved recovery (Hb ≥ 11.0 g/dl) by the end of the intervention, while 13.6% remained in the mild anemia category and 8.1% showed no improvement. In the mild anemia group, 88.6% recovered, 9.1% remained in the mild anemia category, and 2.3% worsened to moderate anemia. Among children with borderline normal hemoglobin at baseline, 95.0% maintained or improved their status to full recovery, while 4.3% declined to mild anemia and 0.7% to moderate anemia. Notably, even in the most severe group at baseline, nearly four out of five children achieved hemoglobin levels ≥ 11.0 g/dl after 3 months, underscoring the overall effectiveness of the dehydrated bovine blood intervention regardless of initial anemia severity. The small proportion of children who showed worsening across all groups—particularly the 2.3% in the mild anemia group and 0.7% in the borderline normal group who declined to moderate anemia—may reflect individual variability in absorption, compliance, or concurrent health conditions that warrant further investigation (Table 6, Figure 3).

**Table 6:** Transition matrix of hemoglobin classification from baseline to month 3.

Baseline classification	Month 3 classification			Total *n* (%)
	Moderate anemia (Hb<10.0 g/dl)	Mild anemia (Hb 10.0–10.9 g/dl)	Recovered (Hb ≥11.0 g/dl)	
Moderate anemia (Hb 7.0–9.9 g/dl)	22 (8.1%)	37 (13.6%)	**213 (78.3%)**	**272 (100%)**
Mild anemia (Hb 10.0–10.9 g/dl)	22 (2.3%)	85 (9.1%)	**830 (88.6%)**	**937 (100%)**
Borderline normal (Hb 11.0–11.5 g/dl)	39mm7 (0.7%)	44 (4.3%)	**961 (95.0%)**	**1,012 (100%)**
**Total**	**51 (2.3%)**	**166 (7.5%)**	**2,004 (90.2%)**	**2,221 (100%)**

Baseline classification uses the study diagnostic thresholds: moderate anemia Hb 7.0–9.9 g/dl; mild anemia Hb 10.0–10.9 g/dl; borderline normal Hb 11.0–11.5 g/dl; Month 3 classification: recovered = Hb ≥11.0 g/dl (borderline normal and normal values combined).

Values represent n (row %).

Bold cells indicate proportions ≥ 30%.

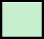
: improved classification (Hb increased to a better category); 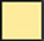: unchanged classification (same category at baseline and month 3); 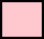: worsened classification (Hb decreased to a worse category.

## 4 Discussion

The WHO recommends that, for the prevention of anemia, iron supplementation should be administered for 3 consecutive months, varying from 10 to 12.5 mg of elemental iron in children aged 6 to 23 months or from 40 to 42.5 mg of iron in children aged 24 to 59 months. Recent evidence indicates that treatment regimens providing approximately 65 mg of elemental iron (three times a day) and 40 mg (two times a day) are associated with increased hemoglobin in children with anemia (19). Likewise, it can be stated that the consumption of 11 mg per day of dehydrated heme iron in children aged 6 to 35 months consumed for 3 to 4 months is associated with improvements in the prevention and recovery from anemia; and having a hemoglobin level above normal values has been associated with improved (17) psychomotor skills in children who previously had anemia (20).

According to intervention studies, polysaccharide iron complex (which has a better taste) has the highest adherence, but at the same time, the increase in hemoglobin in children who consume it is lower than with ferrous sulfate (despite adverse reactions such as vomiting being reported at the time of consumption) (21), in contras, in this study, children aged 24–35 months with moderate anemia who consume dehydrated bovine blood showed higher recovery and a hemoglobin increase of 44.8% (22), as found in this study, children aged 24 to 35 months are 4.25 times more likely to recover. The adverse reactions to consuming iron supplements are diverse, such as: diarrhea, vomiting, abdominal pain, constipation (21), whereas no adverse reactions or difficulties at the time of consumption were spontaneously reported during this study's monitoring activities. This higher recovery in children aged 24 to 35 months may be related to better food acceptance at this age, which facilitates consumption of the dehydrated blood mixture.

In another study, where cookies fortified with bovine blood were consumed, an increase in hemoglobin of 1.2 mg/dl was evidenced, going from 10.4 mg/dl to 11.7 mg/dl (23), and where canned bovine blood was consumed, it increased an average of 0.95 mg/dl (24). For this study, hemoglobin in children increased from 7.1 to 14.4 mg/dl respectively in 3 months of intervention.

Research indicates that children with the highest prevalence of anemia in urban areas are those aged 6 to 11 months (25), which differs from this study that found a higher diagnosis of anemia in children aged 12 to 23 months, and this is often due to not consuming a balanced diet that includes foods with heme iron, parasitosis, mothers with low educational levels (26), mothers who work in the private sector (25), and being scarce due to the type of microcytic, normocytic, and macrocytic anemia (27). In another study, it was found that children with iron deficiency anemia were males (28) and those with greater adherence to iron treatment were those in the moderate anemia group (29), information that is consistent with what was found in this research, but different from Flores (26) who found the highest prevalence of anemia in girls. It was also found that most children have mild anemia and consume on average iron-rich foods four times a week (24), data that are consistent with what was found in this research, where children consume dehydrated blood an average of 4.6 times a week.

## 5 Conclusion

Evidence demonstrates, first, that the cause of anemia is multifactorial, with iron deficiency anemia being the most common but not the only one; and as treatment, the administration of ferrous sulfate according to age is used, which can be drops or syrup with a dose of 3 mg/kg/day in children under 35 months (17). And due to the adverse reactions of ferrous sulfate, the consumption of chicken blood, bovine blood, etc. is promoted, finding that hemoglobin recovery/maintenance reached 96.3% among children aged 24 to 35 months, the age group with the highest rate overall (Table 3). This higher recovery may be related to the direct engagement with feeding practices carried out with the mothers and/or caregivers of children diagnosed with anemia, which increased their level of knowledge about feeding their children.

The limitations of the study were that the families of children with anemia lived far from the health facility and did not have sufficient financial resources to attend all the educational sessions scheduled by the trained professional.

Additionally, this study employed a single-group, pre-post design without a control group, which limits causal inference regarding the effectiveness of dehydrated bovine blood consumption. Observed improvements in hemoglobin could have been influenced by concurrent factors such as changes in dietary intake, co-administration of other supplements, resolution of subclinical infections, or regression to the mean, particularly among children with the lowest baseline hemoglobin values. This study did not collect data on several variables known to influence hemoglobin levels in young children, including anthropometric and nutritional status, concurrent iron or multiple micronutrient supplementation, intestinal parasitic or other infections, breastfeeding status, household socioeconomic conditions, number of children per household, and the degree of dietary control over the child's intake beyond the supplement provided. The absence of these covariates precluded statistical adjustment for potential confounding, and the observed associations between dehydrated bovine blood consumption and hemoglobin recovery should therefore be interpreted with caution.

Although a structured field-monitoring system (daily tracking cards and a caregiver support group) was implemented to support consumption, adherence in this study was estimated indirectly through hemoglobin response rather than through a directly quantified, individual-level measure of dehydrated blood intake; this outcome-based approach does not allow distinguishing true non-consumption from non-response to consumption despite adequate intake. Adverse effects were not systematically monitored using a structured tool, so the absence of spontaneous reports should not be interpreted as definitive evidence of the product's safety. The specific manufacturing process and iron content per 100 g of the dehydrated bovine blood product were not independently verified by the research team, as this information was not available from the supplier at the time of this analysis.

Finally, this study was conducted exclusively within health facilities of DIRIS Lima Centro in the district of San Juan de Lurigancho, an urban setting in metropolitan Lima; consequently, the generalizability of these findings to rural areas or other regions of Peru with differing anemia prevalence and food security profiles (e.g., the Andean highlands), or to other countries, may be limited. Future studies should incorporate a control or comparison group, collect data on the unmeasured covariates noted above, directly quantify consumption and adverse effects using structured tools, report the complete nutritional and microbiological characterization of the dehydrated blood product used, and span diverse geographic and sociocultural settings to confirm the generalizability and causal interpretation of these findings.

The findings found in this research will help improve public policies for the reduction of anemia by using dehydrated bovine blood as a nutritional supplement, this being low cost, easily accessible, and having high adherence. Likewise, this study will help to continue investigating the use of dehydrated bovine blood with a Hazard Analysis and Critical Control Point (HACCP) plan in pregnant women and patients with other types of anemia diagnoses.

## Data Availability

The original contributions presented in the study are included in the article/supplementary material, further inquiries can be directed to the corresponding authors.
